# 1223. *In Vitro* Activity of Ceftazidime-Avibactam and Comparator Agents Against Enterobacterales from ICU and Non-ICU Wards Collected in Latin America and Globally as part of the ATLAS Surveillance Program 2017-2019

**DOI:** 10.1093/ofid/ofab466.1415

**Published:** 2021-12-04

**Authors:** Sibylle Lob, Meredith Hackel, Gregory Stone, Daniel F Sahm

**Affiliations:** 1 IHMA, Inc., Schaumburg, IL; 2 Pfizer, Inc., Groton, CT

## Abstract

**Background:**

Ceftazidime-avibactam (CAZ-AVI) is a β-lactam/non-β-lactam β-lactamase inhibitor combination with activity against Enterobacterales producing class A, C and some class D β-lactamases. Resistance caused by these β-lactamases is especially high in ICUs. This study evaluated the *in vitro* activity of CAZ-AVI and comparators against Enterobacterales isolates from patients in ICU and non-ICU wards.

**Methods:**

Non-duplicate clinical isolates were collected in 2017-2019 from patients in Asia/Pacific, Europe, Latin America, and Middle East/Africa. Susceptibility testing was performed using CLSI broth microdilution and interpreted using CLSI 2021 and FDA (tigecycline) breakpoints. PCR and sequencing were used to determine the β-lactamase genes present in all isolates with meropenem (MEM) MIC >1 µg/ml, and *Escherichia coli*, *Klebsiella* spp. and *Proteus mirabilis* with aztreonam or ceftazidime MIC >1 µg/ml.

**Results:**

The activity of CAZ-AVI and comparators is shown in the table. Susceptibility rates among global Enterobacterales were generally lower for isolates from patients in ICU than non-ICU wards, but this difference was small for CAZ-AVI, which inhibited >96% of isolates from both ward types. Among MEM-nonsusceptible (NS) isolates, CAZ-AVI was active against 62.3% and 65.6% of ICU and non-ICU isolates, respectively, and 36.3% and 33.2%, respectively, carried metallo-β-lactamases (MBLs). CAZ-AVI inhibited >97% of MEM-NS MBL-negative isolates collected globally. Antimicrobial activity against all Enterobacterales from both ICU and non-ICU wards in Latin America (LA) was generally similar to the global average. Among MEM-NS isolates, antimicrobial activity of CAZ-AVI was >10 percentage points higher in LA than the global average among isolates from both ward types, at least partly because of a lower proportion of MBL-positive isolates in this subset (24.4% and 22.0% in ICU and non-ICUs, respectively). CAZ-AVI inhibited >98% of MEM-NS MBL-negative isolates from LA.

Results Table

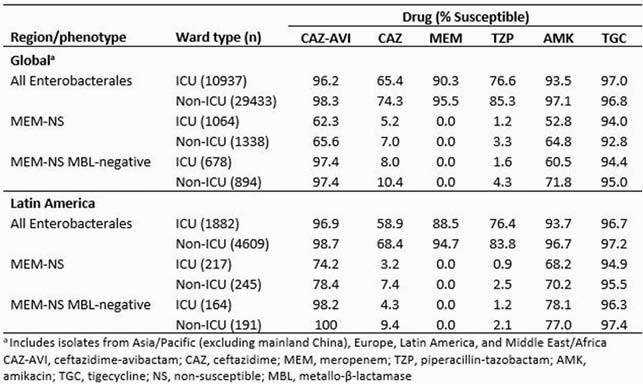

**Conclusion:**

CAZ-AVI provides a valuable treatment option for infections caused by Enterobacterales that do not carry MBLs, including those from patients in ICU wards, where antimicrobial resistance is typically higher.

**Disclosures:**

**Sibylle Lob, PhD**, **IHMA** (Employee)**Pfizer, Inc.** (Independent Contractor) **Meredith Hackel, PhD MPH**, **IHMA** (Employee)**Pfizer, Inc.** (Independent Contractor) **Gregory Stone, PhD**, **AztraZeneca** (Shareholder, Former Employee)**Pfizer, Inc.** (Employee) **Daniel F. Sahm, PhD**, **IHMA** (Employee)**Pfizer, Inc.** (Independent Contractor)

